# Increasing GSH-Px Activity and Activating Wnt Pathway Promote Fine Wool Growth in FGF5-Edited Sheep

**DOI:** 10.3390/cells13110985

**Published:** 2024-06-05

**Authors:** Xue-Ling Xu, Su-Jun Wu, Shi-Yu Qi, Ming-Ming Chen, Zhi-Mei Liu, Rui Zhang, Yue Zhao, Shun-Qi Liu, Wen-Di Zhou, Jin-Long Zhang, Xiao-Sheng Zhang, Shou-Long Deng, Kun Yu, Yan Li, Zheng-Xing Lian

**Affiliations:** 1 Beijing Key Laboratory for Animal Genetic Improvement, National Engineering Laboratory for Animal Breeding, Key Laboratory of Animal Genetics and Breeding of the Ministry of Agriculture, College of Animal Science and Technology, China Agricultural University, Beijing 100193, China; xuelingxu@fafu.edu.cn (X.-L.X.); amywsj@cau.edu.cn (S.-J.W.); bs20223040372@cau.edu.cn (S.-Y.Q.); chenmingming1937@cau.edu.cn (M.-M.C.); s20193040539@cau.edu.cn (Z.-M.L.); zhaoyuetx@cau.edu.cn (Y.Z.); liusq28@126.com (S.-Q.L.); b20183040325@cau.edu.cn (W.-D.Z.); 2 College of Bee Science and Biomedicine, Fujian Agriculture and Forestry University, Fuzhou 350002, China; 3 Academy of Military Medical Sciences, Beijing 100071, China; zr123@cau.edu.cn; 4 Institute of Animal Husbandry and Veterinary Medicine, Tianjin Academy of Agricultural Sciences, Tianjin 300381, China; jlzhang1010@163.com (J.-L.Z.); zhangxs0221@126.com (X.-S.Z.); 5 Institute of Laboratory Animal Sciences, Chinese Academy of Medical Sciences and Comparative Medicine Center, Peking Union Medical College, Beijing 100005, China; dengshoulong@cnilas.org

**Keywords:** *FGF5*, secondary hair follicle, proliferation, cortisol, antioxidant, Wnt

## Abstract

Fibroblast growth factor 5 (*FGF5*) plays key roles in promoting the transition from the anagen to catagen during the hair follicle cycle. The sheep serves as an excellent model for studying hair growth and is frequently utilized in various research processes related to human skin diseases. We used the CRISPR/Cas9 system to generate four *FGF5*-edited Dorper sheep and only low levels of FGF5 were detected in the edited sheep. The density of fine wool in GE sheep was markedly increased, and the proportion of fine wool with a diameter of 14.4–20.0 μm was significantly higher. The proliferation signal in the skin of gene-edited (GE) sheep was stronger than in wild-type (WT) sheep. *FGF5* editing decreased cortisol concentration in the skin, further activated the activity of antioxidant enzymes such as Glutathione peroxidase (GSH-Px), and regulated the expression of Wnt signaling pathways containing Wnt agonists (Rspondins, Rspos) and antagonists (Notum) in hair regeneration. We suggest that *FGF5* not only mediates the activation of antioxidant pathways by cortisol, which constitutes a highly coordinated microenvironment in hair follicle cells, but also influences key signals of the Wnt pathway to regulate secondary hair follicle (SHF) development. Overall, our findings here demonstrate that *FGF5* plays a significant role in regulating SHF growth in sheep and potentially serves as a molecular marker of fine wool growth in sheep breeding.

## 1. Introduction

The hair follicles are primary accessory organs of the skin, regulating hair growth and shedding [1]. In most mammals, the hair follicle cycle is the same and divides into three main phases of anagen, catagen, and telogen [2]. Wool, especially fine wool, has many properties such as stain resistance, softness, and warmth, which are of high economic value in the sheep industry. Fiber diameter is a key trait in wool sheep breeding and is closely related to the morphogenesis and development of hair follicles in sheep skin. Therefore, studying and understanding the molecular genetic mechanisms involved in the formation of wool traits will help accelerate the breeding of fine wool sheep. In addition, the occurrence and growth of sheep hair follicles are similar to those in the human head and are independent of neighboring hair follicles. Thus, sheep are considered as an additional animal model with potential for understanding skin-relevant diseases in humans, especially in terms of hair growth.

The fibroblast growth factor 5 (*FGF5*) gene belongs to the FGF family and mediates FGF5 signaling by binding to FGFR1 and forming a ternary complex with heparan sulfate proteoglycans (HSPGs) [3]. Notably, FGF5 is a hair growth inhibitor that is highly expressed in the late anagen phase [4,5]. FGF5 was originally identified in angora mice, which carry a spontaneous mutation of the *FGF5* gene [6,7]. Angora mice had a prolonged anagen phase, an abnormally increased proportion of anagen hair follicles, and longer hair than wild-type (WT) mice. FGF5-deficient or knockout (KO) animals (including mice, sheep, cats, dogs, donkeys, guinea pigs, and even humans) have also been shown to have abnormal hair length [8,9,10,11]. In addition, previous studies have shown that base pair editing of the *FGF5* gene in goats induces a C-T transition and an increase in wool fibers [12]. In our previous study, we constructed *FGF5*-edited heterozygous sheep by injecting Cas9 mRNA and single-guide RNA (sgRNA) directly into zygotes using the CRISPR/Cas9 system, and demonstrated that the *FGF5* editing increased wool length and density. However, the exact role of FGF5 in hair follicle development is unknown, and it is currently worth investigating whether it has effects other than promoting hair follicle cycle alteration.

Stress can trigger and aggravate hair loss, for example through alopecia areata (AA), telogen effluvium, and androgenetic alopecia. Stress increases apoptotic cells, inhibits hair bulb keratinocyte proliferation, alters the hair cycle, and induces premature regression. It has been found that the skin has the ability to produce glucocorticoids (GCs, primarily cortisol) as a response to stress. GCs are an important class of steroid hormones that encompass a variety of biological processes, including vertebrate development, metabolism, inflammation, and programmed cell death [13,14]. Natural GC and its synthetic analogs act via the glucocorticoid receptor (GR). On ligand binding, GR undergoes a conformational change and translocates to the nucleus, where it regulates gene expression by binding directly to DNA or by interacting with other transcription factors. Classical reports have shown that dexamethasone (synthetic GR agonist, Dex) injection in mice leads to retarded hair growth and hair loss. Consistent with these observations, in ectoderm-targeted transgenic mice overexpressing the GR [keratin 5 (K5)-GR mice], increased GR levels resulted in reduced hair follicle numbers and abnormal hair follicle morphology [15]. An animal model demonstrated that administration of dexamethasone-21-acetate (0.1%) to depilation-induced growing follicles (growth phase VI) on the back of mice could induce the hair follicle regression catagen phase [16]. In fact, both men and women with androgenetic alopecia have elevated cortisol levels. Interestingly, previous studies have suggested that the *FGF5* gene may be involved in the pathogenesis of androgen alopecia [17]. However, it is unclear whether cortisol is involved in wool growth and hair follicle development processes in *FGF5* gene-edited (GE) sheep.

In the present study, we explored wool production performance and secondary follicle development in *FGF5* GE sheep. The results showed that FGF5 was associated with high-quality fine wool growth in sheep. The potential mechanism of FGF5 and cortisol in secondary follicle development was further explored. *FGF5* editing may reduce cortisol, a hormone that inhibits secondary hair follicle (SHF) initiation and maturation. Therefore, we hypothesized that *FGF5* editing may promote SHF growth by reducing cortisol in the skin, which in turn increases antioxidant enzyme activity and Wingless-type (Wnt) signaling.

## 2. Materials and Methods

### 2.1. Ethics Statement

All procedures performed for this study were consistent with the National Research Council Guide for the Care and Use of Laboratory Animals, and ethics/project approval was granted from the Animal Care and Use Committee at China Agricultural University (approval number AW81012202-1-1).

### 2.2. Animals

**Figure 1:**
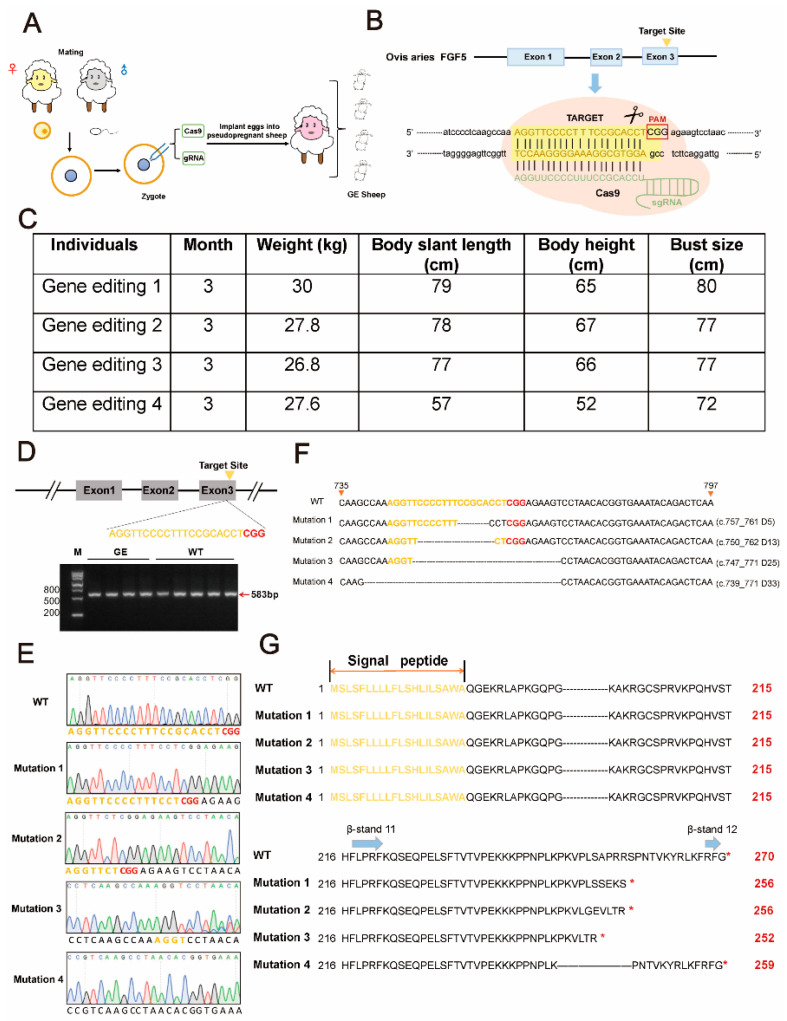
*FGF5* knockout sheep were obtained by CRISPR/Cas9. (**A**) Schematic diagram of the experimental design. Cas9 mRNA and sgRNA were co-injected into single-cell embryos; the embryos were then implanted into the uterus of a third animal. The editing results of lambs were detected after birth. (**B**) Schematic diagram of the structure and sgRNA targeting site of the sheep *FGF5* gene. Three coding exons and intron sequences are shown in blue boxes and black lines, respectively. The sgRNA-targeting sequence is highlighted in yellow, and the PAM sequence is presented in red. (**C**) The body size information of GE sheep. (**D**) Identification of GE sheep and partially WT sheep by PCR assay. M, 2 kb DNA ladder marker. (**E**) Sequencing map of the edited *FGF5* loci detected in GE sheep. (**F**) InDel forms around PAM for GE sheep and the targeting locus of sgRNA: Cas9. The truncated sequences resulting from the deletion of c.757_761del (D5), c.750-762del (D13), c.747_771del (D25), and c.739_771del (D33) are four predominant targeted events. sgRNA-targeting sites are shown in yellow text; PAM sequences are marked in red; InDels of clone sequencing in individuals are shown to the right of the sequence. (**G**) Alignment of amino acid sequences of FGF5 in GE and WT proteins. Protein AA sequences are presented in black text; signal peptides are shown in yellow; the secondary structure of β-strands is emphasized in blue. * indicates termination.

The production process of four *FGF5* GE sheep is presented in Figure 1A. Cas9 mRNA and Ovis aries *FGF5* sgRNA transcription templates were previously generated in our laboratory. A schematic structure of the sheep *FGF5* gene and the target site of *FGF5* sgRNA are shown in Figure 1B.

For DNA sequencing of microinjected individuals, the genomic DNA was extracted from sheep blood. PCR amplification was performed by primers using 50 ng/uL of DNA as template. The primers sequenced were as follows: *FGF5*-F, TGCAAGTTCAGGGAGCGATT and *FGF5*-R, ATCCCTGTATGCACCAAGCA. The types of mutations were determined by comparing the sequenced alleles with the WT allele. The WT group included siblings of the same age as the GE group.

### 2.3. RNA Extraction and Quantitative RT-PCR Analysis

RNA was extracted using an RNA extraction kit (Aidlab, Beijing, China) and converted to cDNA with the PrimeScript RT kit (TaKaRa, Kyoto, Japan) according to the manufacturer’s protocol. Quantitative RT-PCR was performed in the Mx3000P instrument (Agilent Technologies, Santa Clara, CA, USA) using the SYBR Premix Ex Taq II kit (TaKaRa, Kyoto, Japan). The data were normalized using housekeeping gene GAPDH and the relative gene expression levels were calculated using the 2^−ΔΔCt^ method. The PCR primer sequences, including gene-specific primers for the *FGF5*-edited regions, are shown in Supplementary Table S1. The data were normalized using GAPDH and relative gene expression levels were calculated using the 2^−ΔΔCt^ method.

### 2.4. ELISA and Western Blot

For ELISA, skin tissue samples of the GE and WT groups were prepared and homogenized by grinders, then centrifuged at 13,000× *g* to precipitate the insoluble materials. FGF5 and cortisol concentrations in the skin were determined using the FGF5 ELISA kit (TSZELISA, Framingham, MA, USA) and the cortisol ELISA kit (Mlbio, Shanghai, China) in accordance with the manufacturer’s protocol.

For Western blotting, skin tissues were subjected to total protein extraction using Minute^TM^ Total Protein Extraction Kit for Skin Tissue (Invent Biotechnologies, Beijing, China) and then quantified using the BCA assay. Sheep skin tissue extracts containing equal amounts of protein were separated on 12% SDS-polyacrylamide gel (SDS-PAGE). The proteins in the gel were then transferred to polyvinylidene difluoride membrane (PVDF), blocked with 5% skim milk for 1 h, and then incubated with anti-FGF5 (Thermo Fisher Scientific, Waltham, MA, USA), anti-GR (Santa cruz, Dallas, TX, USA), anti-Ki67 (Beyotime, Shanghai, China), anti-Bcl2 (Proteintech, Wuhan, China), anti-BAX (Abmart, Shanghai, China), anti-GAPDH (Sangon Biotech, Shanghai, China), and Beta tubulin (Proteintech, Wuhan, China) overnight at 4 °C. After washing the blot was incubated with secondary antibodies. The blot was detected using a chemiluminescence substrate (Thermo Fisher Scientific, Waltham, MA, USA) and images of the blot were captured using a chemiluminescence system (BIO-RAD, Hercules, CA, USA) and analyzed using the ImageJ software v.1.52 (National Institutes of Health, Bethesda, MD, USA).

### 2.5. Wool Phenotype Analysis

We analyzed the diameter of 200 randomly selected fibers using an automated analyzer (CU-6, Beijing United Vision Technology, Beijing, China). Based on the presence or absence of medulla, wool was classified as non-medullated wool, heterotypical wool, or medullated wool. According to the diameter of the unmyelinated wools, the non-medullated wools were divided into three types: 14.4–20.0 μm, 20.1–25.0 μm, >25.1 μm.

Cashmere fiber density was determined by recording photographs within 48 h after shaving. The photographs were taken with a handheld digital microscope (Hot Beauty Skin Tester Ht-B20S). The scale was used to correct the actual field of view and calculate the density of the coarse and fine wool, respectively. For each part (anterior shoulder, body side, hindquarter), this was repeated three times for both experimental and control individuals.

Wool samples containing coarse wool and fine fibers were weighted on a precision electronic balance, then washed with carbon tetrachloride and warm water, air dried in a fume hood, and weighed again. Fiber lengths were determined by placing the zero end of a steel ruler firmly on one end of the fiber and gently stretching the fiber until all bends and kinks disappeared before determining the length. The wool length of each sample was taken as the average of 100 fiber lengths. The clean wool yield, wool grease rate, and stretched length were measured manually in accordance with the National Sheep Wool Quality Inspection Standards of China (GB1523-2013). All these experiments were repeated more than three times for the GE and WT group.

### 2.6. Histology Staining of Skin Tissues

Four skin samples from the GE group were collected at the shoulder position. After fixation with Bouin’s fixative and paraffin embedding, skin samples were cut into 2 μm slices and stained with hematoxylin and eosin (H&E) staining to determine the traits of the hair follicles. The slices were used to calculate the ratio of SHFs and the ratio of secondary to primary follicles (S/P). For each counting, hair follicles at the top and left edge were counted, while anything that was cut at the bottom and right edge was not included. Depending on the size, arrangement position, and accessory structure, primary hair follicles (PHFs) and SHFs can be easily distinguished.

### 2.7. Measurement of Skin Antioxidant Enzyme Activity

The skin tissues collected after hair removal were cut into small pieces and ground for further disintegration. Total antioxidative capacity (T-AOC) was evaluated using the FRAP method and catalase (CAT) activity was measured by the ammonium molybdate method. The above enzyme assay kits were purchased from Nanjing Jiancheng Bioengineering Institute (Nanjing, China). Glutathione peroxidase (GSH-Px) activity was quantified by NADPH method (Beyotime). The absorbance was measured at the specified wavelength in strict accordance with the manufacturer’s instructions.

### 2.8. Isolation and Analysis of DPCs

To isolate dermal papilla cells (DPCs), the shoulder skin of the GE and WT groups was excised and the subcutaneous fat tissue removed using a surgical scalpel. The skin was floated in neutral protease (Gibco, Grand Island, NE, USA) at 37 °C for 2 h and then the epidermis was slowly peeled off. Then skin was digested with Collagenase II (Gibco, Grand Island, NE, USA) for 30 min at room temperature to remove excess tissue between hair follicles. Most DPCs were exposed at the hair follicle bulb and then DPCs were dissected microscopically. Finally, the isolated DPCs were planted in the culture dish. DPCs were cultured in Dulbecco’s Modified Eagle Medium/Nutrient Mixture F-12 (DMEM/F-12) containing 20% fetal bovine serum (FBS) supplemented and 1% penicillin/streptomycin (Invitrogen, Carlsbad, CA, USA). Cells were stained with the following antibodies: mouse anti-SMA (1:100, Abcam, Cambridge, UK) and rabbit anti-CD133 (1:100, Abnova, Walnut, CA, USA) at 4 °C overnight. The DPCs were identified as the SMA^+^-CD133^+^ population as previously described. In all experiments, cells were used for up to 10 generations. All experiments were performed in quadruplicate.

### 2.9. DPCs Proliferation

Cell proliferation was assayed using cell counting kit-8 (CCK-8; MCE, NJ, USA). DPCs were seeded and cultured in 96-well plates at a concentration of 5000 cells per well. At the different time points of 0, 24, 48, and 72 h, 100 mL of fresh medium containing 10 μL CCK-8 reagent was added to each well. After incubation at 37 °C for 2 h, absorbance was measured at 450 nm using a microplate reader.

The proliferation of DPCs was measured using the EdU assay. The DPCs (5000 cells/well) were cultured in a 96-well plate with DMEM/F-12. After incubation with the 50 μM EdU dye (100 μL/well) for 2 h, the supernatant was removed, and DPCs were fixed for 30 min in 4% paraformaldehyde at room temperature. We added 50 μL of 2 mg/mL glycine per well, incubated for 5 min on a decolorized shaker, washed three times with PBS, and incubated with hoechst33342 protected from light, after which images were acquired using the confocal microscope (Nikon A1, Tokyo, Japan).

### 2.10. Statistics

Statistical analysis was performed in GraphPad Prism 8 (GraphPad Prism software, La Jolla, CA, USA). Data on fiber density and fiber weight of GE and WT animals were compared by one-way ANOVA. Data are expressed as the mean ± SEM. The difference was summarized as significant at *p* < 0.05. Results were based on at least three replicates and all the groups were examined as independent measurements.

## 3. Results

### 3.1. Generation of FGF5 Gene-Edited Sheep

A sgRNA-targeting exon 3 of *FGF5* gene was previously designed in our laboratory and utilized for in vitro embryo targeting [18]. Four *FGF5*-edited Dorper sheep were successfully obtained by injecting sgRNA/Cas9 into the cytoplasm of embryos, and the edited sites are shown in Figure 1A,B (including three females and one male). The GE sheep obtained were in good body condition with no abnormalities (Figure 1C). The 12 most likely off-target sites have been predicted in the sheep genome, and no InDels have been detected [17].

The blood DNA templates of positive individuals were amplified and sequenced and, not surprisingly, four different mutations appeared (Figure 1D,E). Each GE individual was further analyzed by T-A clone sequencing, and various modifications were accurately described. In all transgenic sheep, the four modifications were c.757_761del (D5), c.750_762del (D13), c.747_771del (D25), and c.739_771del (D33). In all GE sheep, three frameshift mutations and one in-frame deletion were identified (Figure 1F). The InDels result in AA deletion or frameshift of FGF5 protein. The secondary structure of FGF5 contains 12 conserved β-strand structures, which are the functional regions of FGF5. The critical β-12 strands were destroyed or altered, leading to premature termination of FGF5 function, and thus complete or partial abolition of FGF5 function (Figure 1G). This suggests that knockout of *FGF5* by CRISPR/Cas9 can effectively produce loss-of-function modifications.

### 3.2. The Expression of FGF5 mRNA and Protein Was Decreased in GE Sheep

**Figure 2:**
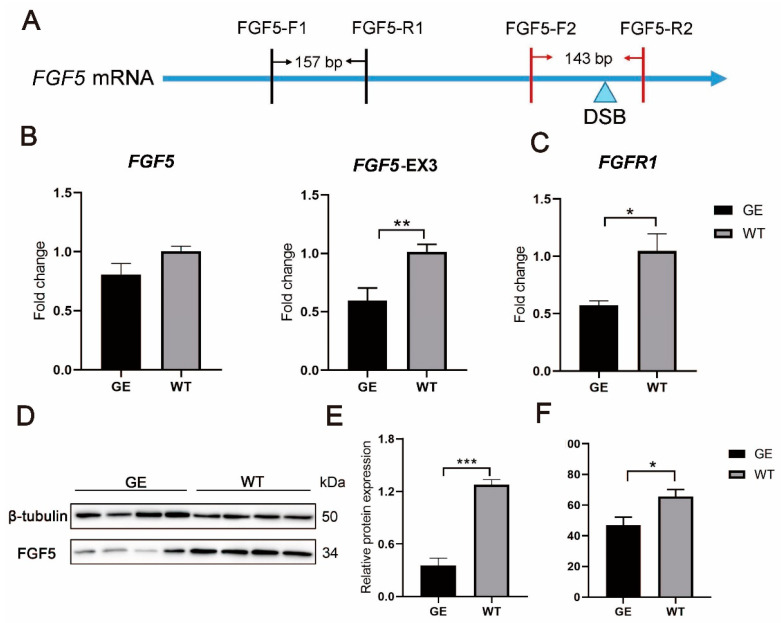
Changes in *FGF5* mRNA and protein level in GE sheep. (**A**) Schematic diagram of *FGF5* gene region amplified by primers. (**B**) Expression of total *FGF5* transcripts (left) and mutated *FGF5* transcripts (right) was determined by RT-qPCR. (**C**) RT-qPCR analysis of FGFR1 transcripts in the skin. (**D**) FGF5 protein levels were determined by Western blot. (**E**) The expression of the FGF5 protein in the skin. (**F**) FGF5 protein levels were determined by ELISA. The protein expression of FGF5 in the GE group was decreased compared with WT in anagen. * *p* < 0.05, ** *p* < 0.01, *** *p* < 0.001.

The total RNA of skin tissues from GE and control sheep was extracted and reverse transcribed to obtain *FGF5* cDNA. Different primers were used to amplify different compartments of *FGF5* cDNA: F1 primer detected total *FGF5* mRNA, F2 primer was designed according to the mutation site, and the 3′ end of the R2 primer was designed in the InDel region of *FGF5* (Figure 2A). The results showed that there was no significant difference in mRNA expression between the GE sheep and the control sheep at the F1 position, but at the F2 site, *FGF5* mRNA was significantly downregulated in the GE group compared with the WT group (Figure 2B). FGFR1 is a high-affinity receptor for FGF5, which inhibits hair growth by blocking the activation of dermal cells through binding to FGFR1 [19]. Correspondingly, FGFR1 expression was also significantly decreased in the GE group (Figure 2C). Moreover, the FGF5 protein was also significantly downregulated by WB and Elisa assays, further indicating the successful disruption of the *FGF5* gene (Figure 2D–F).

### 3.3. Effect of FGF5 Editing on Wool Traits in Sheep

**Figure 3:**
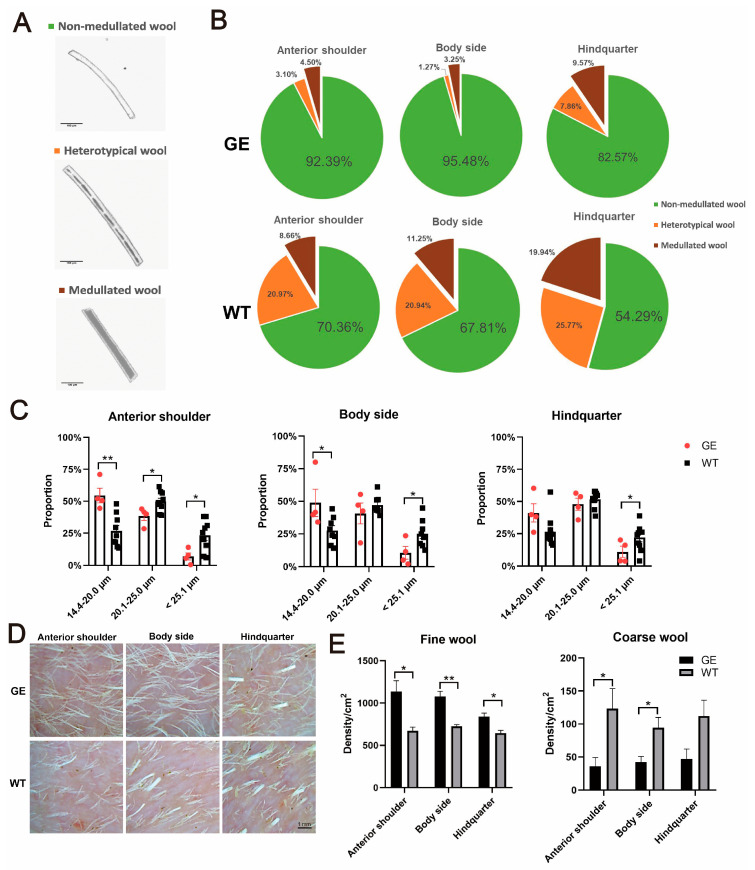
Statistical analysis of wool traits in GE sheep and WT sheep in anagen. (**A**) Photographs of three types of wool from the GE and the WT sheep. (**B**) Proportion of non-medullated wool, heterotypical wool, and medullated wool during anagen phase. (**C**) Proportion of non-medullated wool of different diameters (14.4 µm–20 µm, 20.1 µm–25 µm, and above 25.1 µm) in different positions of GE group and control group. (**D**) Representative images of skin follicles 48 h after shaving. The actual area shown in the figure is 9 mm^2^. (**E**) Statistics of coarse and fine wool densities in different parts of GE and WT groups. The TG sheep showed increased densities of fine wool. The statistical results in Figure (**E**) are from Figure (**D**). * *p* < 0.05, ** *p* < 0.01.

The primary traits contributing to the economic value of sheep wool consist of fiber fineness, density, and length. Therefore, we measured the phenotypic changes in wool caused by the loss of function mutation in FGF5. The fineness and density of the wool were measured from three parts (anterior shoulder, body side, hindquarter) of both the GE and WT group. There were three types of wool in both the GE and control groups, including non-medullated wool, heterotypical wool, and medullated wool (Figure 3A). Analysis of the proportion of wool types in the GE group and the WT group showed that the proportion of non-medullated wool was higher and the proportion of medullated wool was lower in the three parts of the GE group than in the WT group (Figure 3B). The data in Figure 3B were quantified (Figure S1). The proportion of non-medullated wool on the anterior shoulder and body side in the GE group was significantly higher than in the WT group, while the proportion of medullated wool from GE was lowest compared with WT (*p* < 0.05) (Figure S1).

Therefore, we examined the wool fineness of the GE sheep and the control group. The diameters of nonmedullated wool were subdivided into 14.4 µm–20 µm, 20.1 µm–25 µm, and above 25.1 µm. The proportion of wool fineness in the range of 14.4 µm–20 µm on the shoulder and body side of the GE group was significantly higher than WT. Compared to the control group, the proportion of wool fineness in the range of 20.1 µm–25 µm on the shoulder was remarkably decreased in the GE group, as was the proportion of wool fineness above 25.1 µm at the shoulder, body side, and hindquarter (Figure 3C).

Wool density was measured in the GE and WT groups during anagen (Figure 3D). Compared with the WT group, the GE group had a lower density of coarse wool, particularly in the anterior shoulder and body side, whereas the fine wool densities in the GE group were substantially higher than those of WT sheep at all three parts (Figure 3E). Changes in wool density of *FGF5*-edited sheep in telogen were also investigated (Supplementary Figure S2). Compared to WT, the stretch length of fine wool was significantly increased in all three parts of the sheep, as was the stretch length of coarse wool in the rump of FGF5-targeted sheep (Supplementary Figure S2). The above results suggest an increase in yield and quality of the fine wool produced by *FGF5* editing.

Notably, the wool weight of GE sheep was markedly heavier than that of the WT group (Supplementary Figure S4). This indicated that the increase in wool weight is most likely due to an increase in wool length and density. However, as only one month’s worth of data was collected, more data are needed for verification. In addition, we also examined the clean wool yield and wool grease rate of the different parts in GE and control sheep, and there was no significant difference between the GE and control groups (Supplementary Figure S5).

### 3.4. Effect of FGF5 Editing on Hair Follicle Development in Sheep

**Figure 4:**
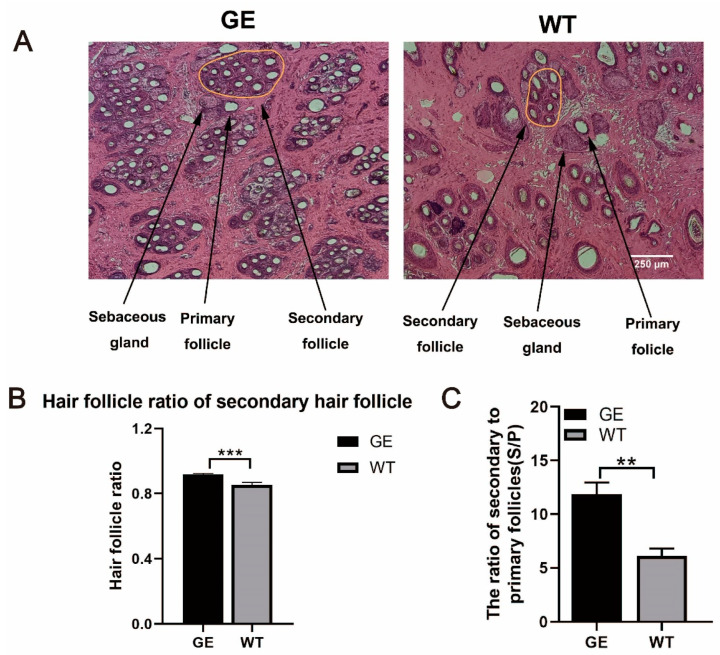
Histological analysis of hair follicle samples in GE sheep and WT sheep in anagen. (**A**) Paraffin sections and H&E staining of skin from GE and WT sheep. The PHFs, SHFs, and sebaceous gland are also labeled with arrows, respectively. (**B**) The total hair follicle ratio of SHFs in the GE and WT group. The proportion of the SHFs in GE was significantly higher than in WT. (**C**) The ratio of S/P in the GE group and the WT group. ** *p* < 0.01, *** *p* < 0.001.

Subsequently, to investigate the effect of *FGF5* editing on the development of sheep hair follicle in anagen, we obtained skin tissue from GE and WT sheep for histological analysis. H&E staining was performed to measure the proportion of SHFs and the ratio of S/P (Figure 4A). The results demonstrated that the proportion of SHFs of GE sheep was obviously increased compared with WT sheep (Figure 4B). Moreover, H&E staining results also indicated that the ratio of S/P was significantly enhanced in GE sheep compared to WT, meaning more fine fibers will be generated in GE sheep (Figure 4C).

Moreover, no apparent changes in the diameter of primary dermal papilla (DP) or the depth of primary follicle and secondary follicles in the skin tissues of GE and WT sheep were observed (Supplementary Figure S6). Overall, the *FGF5*-edited sheep model has long wool, fine wool, and dense hair follicles, which demonstrates that *FGF5*-edited sheep are an ideal model for high-quality sheep wool production.

### 3.5. FGF5 Editing Increases Anti-Apoptotic Signaling in the Skin

**Figure 5:**
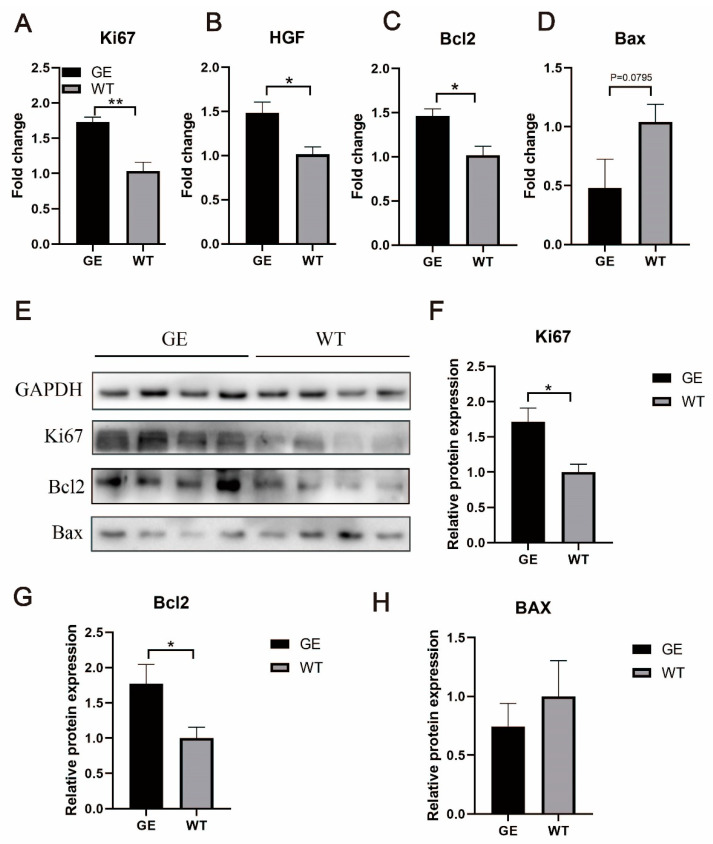
*FGF5* KO decreased the protein expression of pro-apoptotic Bax and stimulated the protein expression of anti-apoptotic Bcl-2 and pro-proliferation Ki67. (**A**) RNA levels of Ki67 in skin tissues were measured by qPCR. (**B**) Expression of HGF gene was determined by qPCR. (**C**) Expression of Bcl2 was quantified by qPCR. (**D**) Expression of Bax was quantified by qPCR. (**E**) Western blotting of Ki67, Bcl2, and Bax proteins in the skin tissue prepared from sheep. The amount of GAPDH was used as a control. (**F**–**H**) Ki67, Bcl2, and Bax protein levels in GE group and WT group. Compared with the WT group, the protein expression levels of Ki67 and Bcl2 in the GE group were significantly changed. * *p* < 0.05, ** *p* < 0.01.

To explore the possible role of *FGF5* editing on SHF development in the skin, we used RT-qPCR and Western blotting to evaluate the expression of proliferative proteins as well as anti-apoptotic Bcl2 and pro-apoptotic Bax proteins in the skin of the GE and WT groups. The mRNA of *Ki67* and *HGF* levels were upregulated significantly (*p* < 0.05) (Figure 5A,B), and *Bcl2* expression was significantly increased (*p* < 0.05) (Figure 5C) in the GE group compared with WT. While *Bax* was attenuated in the GE group, the difference was not statistically significant compared with the WT group (Figure 5D). The results of Western blotting basically showed the same trend, with the Ki67 level significantly increased in the GE group (Figure 5F). The expression of pro-apoptotic protein Bax was decreased in the GE group, with no significant difference (Figure 5H). However, the expression of Bcl-2 protein was markedly increased in the GE group (Figure 5G). These results suggest that downregulation of FGF5 expression enhances the proliferation of SHFs in the skin of GE sheep, which is associated with the ability to maintain a higher level of Bcl-2/Bax ratio.

### 3.6. FGF5 Editing Reduces Cortisol Concentration in the Sheep Skin

**Figure 6:**
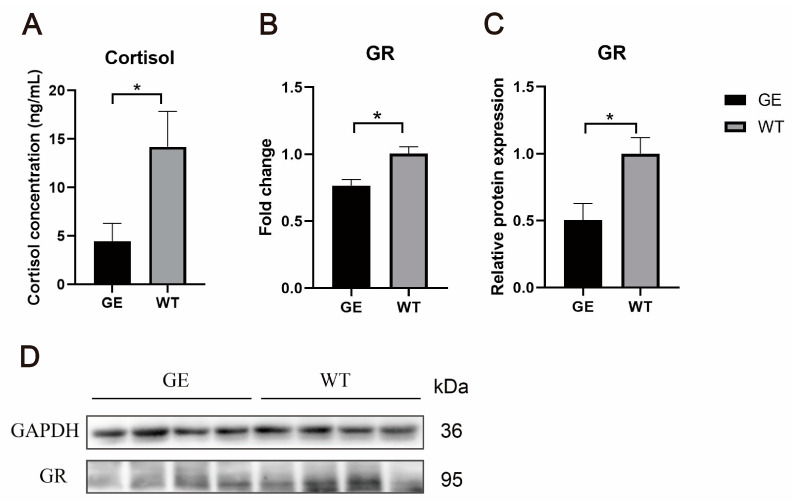
Inhibition of FGF5 promoted a decrease in cortisol concentration in the skin. (**A**) Cortisol levels in the skin. (**B**) The mRNA expression of GR. (**C**) The statistical results of Western blot. (**D**) Western blot analysis of the GR expression. * *p* < 0.05.

*FGF5* editing increased wool length and SHF number in sheep, leading to an increase in fine wools and an increase in the proportion of 14.4 µm–20 µm fine wools. Cortisol has been noted to play a prominent role in inhibiting skin hair follicle development and leading to a reduction in the number of skin hair follicles. Therefore, to investigate the potential mechanism of *FGF5* editing in SHF growth and development, we determined the cortisol concentration and the expression levels of the GR in the skin. The skin cortisol concentration of edited sheep was lower than that of control sheep (Figure 6A), and GR mRNA level in GE group was significantly downregulated compared with the WT group (Figure 6B). Additionally, GR protein levels were remarkably decreased in the GE group (Figure 6C,D). Based on the above results, *FGF5* editing can promote SHF growth by decreasing the gene and protein expression of cortisol and its receptor in the sheep skin.

### 3.7. FGF5 Editing Increases the Activity of Antioxidant Enzymes

**Figure 7:**
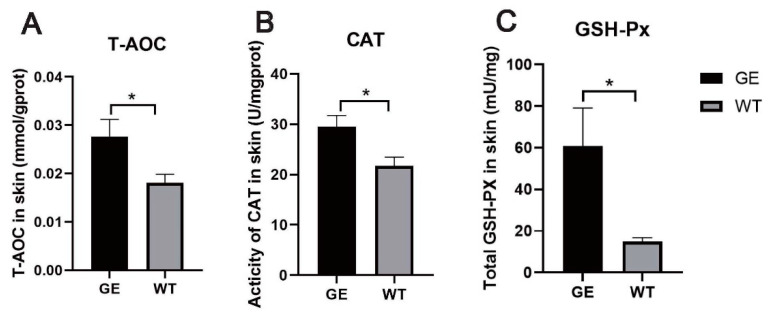
Inhibition of FGF5 promoted an increase in the activity of antioxidant enzymes in sheep skin. The total antioxidant capacity (T-AOC) (**A**), the activity of catalase (CAT) (**B**), and glutathione peroxidase (GSH-PX) (**C**) in the skin of the GE and WT groups. * *p* < 0.05.

High levels of cortisol under stress can lead to oxidative damage, induce hair degeneration, inhibit hair matrix keratinocyte proliferation, and upregulate root sheath cell apoptosis. To determine whether cortisol affects skin antioxidant capacity, total antioxidant capacity and antioxidant enzyme activity in the skin were measured. The T-AOC of skin in the GE group was higher than in the WT group (Figure 7A). Similarly, the loss of FGF5 function in the GE group resulted in a significant increase in skin CAT activity (Figure 7B). Compared with the control group, skin GSH-Px activity was increased in the GE group (Figure 7C).

### 3.8. The Rspondin Protein Family Is Activated by FGF5 Editing

Mammalian hair growth and regeneration are regulated by activation and inhibition of the Wnt signaling pathway in the DP and the hair matrix, which determines the hair growth cycle. Previous studies have shown that FGF5 inhibits the expression of Rspondin (Rspo) in DP by the FGF signaling pathway, thereby terminating the positive feedback loop and thus inducing the catagen phase [5,20]. In addition, the four Rspos that enhance Wnt signaling in WT mice gradually decrease in the middle and late embryonic growth, while the antagonists Notum and DKK2 gradually increase. Therefore, we hypothesized that wool growth mediated by *FGF5* gene editing requires the involvement of the Wnt pathway. We measured the mRNA expression levels for the Wnt signaling pathway using qRT-PCR.

**Figure 8:**
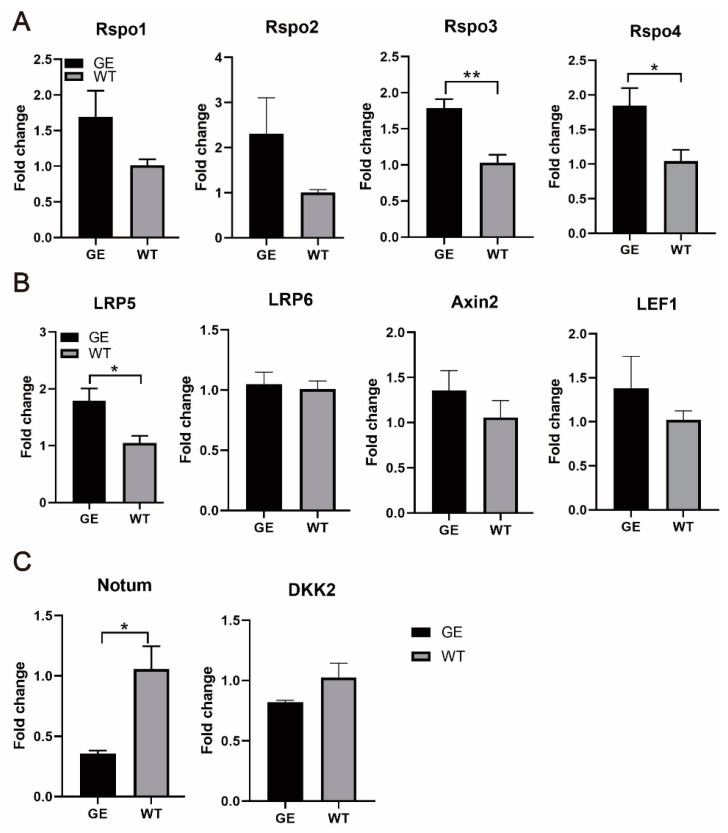
Rspo3 is upregulated in GE sheep. (**A**) Expression of four Rspos in skin tissues of GE and WT groups. (**B**) The mRNA levels of *LRP5*, *LRP6*, *Axin2*, and *LEF1* in the skin tissue. (**C**) Expression of Wnt signaling pathway antagonists was observed using qRT-PCR. Among the 10 Wnt-related genes, eight genes were increased (*Rspo1*, *Rspo2*, *Rspo3*, *Rspo4*, *LRP6*, *LRP5*, *Axin2*, and *LEF1*) and two genes (*Notum* and *DKK2*) were decreased. * *p* < 0.05, ** *p* < 0.01.

Eight genes were increased (*Rspo1*, *Rspo2*, *Rspo3*, *Rspo4*, *LRP6*, *LRP5*, *Axin2*, and *LEF1*) and one gene (*Notum*) was significantly decreased among the 10 Wnt-related genes in the GE group when compared to the WT (Figure 8). Among them, *Rspo3*, *Rspo4*, and *LRP5* levels were significantly increased in the GE group (Figure 8A,B). *DKK2* mRNA level was attenuated in the GE sheep, although no significant difference was observed (Figure 8C). Taken together, these results indicate that *FGF5* editing may stimulate fine wool growth by activating the Wnt signaling pathway.

### 3.9. FGF5 Editing Significantly Promotes Dermal Papilla Cell Proliferation

**Figure 9:**
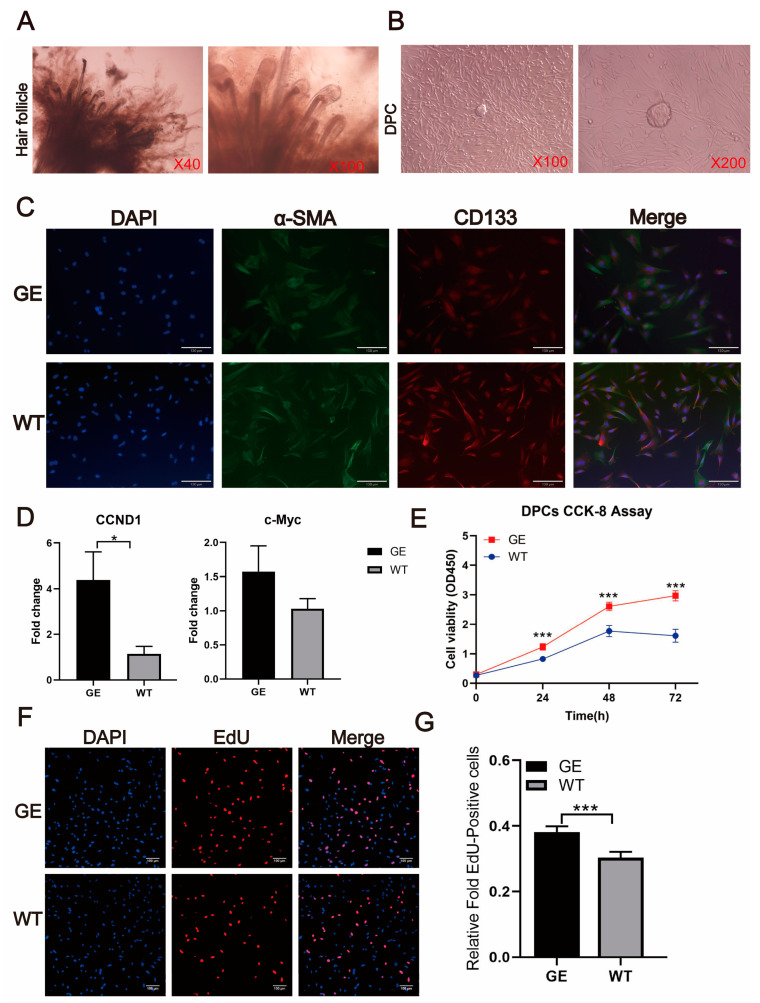
*FGF5* editing increases the proliferation of dermal papilla cells. (**A**) Isolation of dermal papilla cells from sheep SHFs. (**B**) Growth of cells in starburst formation around DP condensates. (**C**) Sheep DPCs were isolated from shoulder skin during the anagen. Immunostaining showed α-SMA (green) and CD133 (red) in the DPCs. DAPI staining (blue) indicates nuclei. (**D**) mRNA expression levels for cell-cycle-related proteins CCND1 and c-Myc. (**E**) The proliferation of DPCs at different times in the GE group and WT group was evaluated by CCK8 assays. (**F**) Confocal microscopy images of changes in the number of edU-positive cells in the GE group and WT group. (**G**) Quantification of EdU cells in the GE group and WT group. * *p* < 0.05, *** *p* < 0.001.

As previously mentioned, DPCs are considered key modulators in HFs, and the proliferation of DPCs is crucial in hair growth and hair cycle processes. We isolated hair papilla cells from skin tissues of the GE and WT groups followed by culturing at 37 °C, 5% CO2 incubator. Cells were readily visible around the condensates. The α-SMA and CD133 proteins in sheep DPCs were analyzed by immunofluorescence techniques (Figure 9A,B). The results of specific markers α-SMA and CD133 expression proved that the cells were DPCs in the anagen stage (Figure 9C).

To investigate the role of *FGF5* in the growth of DPCs, we detected the effects of FGF5 on DPC proliferation. The levels of the cell-cycle-related proteins CCND1 and c-Myc were investigated. As shown in Figure 9D, *CCND1* and *c-Myc* were elevated after *FGF5* editing compared with the control group. According to the OD450 value measured by the CCK-8 assay, the proliferation of DPCs in the GE group was markedly higher than in the WT group (Figure 9E). Next, we validated whether *FGF5* editing promoted the proliferation of DPCs by an EdU incorporation assay (Figure 9F). We observed a significant increase in EdU-positive cells within the GE group compared with the WT group (Figure 9G). Our findings suggest that *FGF5* editing stimulates the proliferation of DPCs by regulating cell-cycle-related proteins. Thus, *FGF5* editing could promote DPC proliferation.

## 4. Discussion

Most reports have claimed that the wool of sheep and goats becomes longer after *FGF5* KO [18,21,22]. In addition, it has been reported that the *FGF5* gene can improve wool density [17,22]. This was also verified in our research. However, there is no specific report on the effect of the *FGF5* gene on wool fineness. Here, we demonstrated that the *FGF5* gene affects wool fineness and SHF density in sheep, and that *FGF5*-edited sheep have more fine hairs and a greater proportion of fine hairs in the 14.4–20.0 μm diameter range.

To explore the beneficial effects of *FGF5* editing on SHF development, we first assessed whether *FGF5* is associated with changes in intrafollicular proliferation and/or apoptosis in skin. The initiation and maturation of secondary follicles is dependent on the balance of apoptosis and proliferation of mesenchymal stem cells and keratinocytes. This study showed that *FGF5* editing increased the levels of the anti-apoptotic protein Bcl-2, attenuated the expression of the pro-apoptotic protein Bax, and upregulated Ki67 expression. Thus, *FGF5* editing may provide additional cytoprotection and promote fine wool growth by inhibiting the rise in Bax/Bcl-2 ratio. Considering that FGF5 is mainly expressed in the outer root sheath, it acts by binding to the FGFR1 in DPCs [23]. *FGF5* editing may work by triggering the proliferation of some vital cells in HFs, like DPCs. We compared the proliferation of hair papilla cells in the GE and control groups and found that *FGF5* editing facilitated the maintenance of hair papilla cell proliferation in vitro and increased C-myc and CyclinD1 expression. It is well known that downstream target genes of Wnt signaling, *C-myc* and *cyclinD1*, participate in cell growth and cell cycle regulation [24,25]. C-myc was required to maintain the stem-cell-like phenotypes of DPCs, a key biological process for maintaining a high rate of hair growth [26,27]. DPCs can interact with the matrix cells and release various cytokines to activate several pathways, helping the matrix cells differentiate into various structures like outer root sheath cells, internal root sheath cells, and keratinocytes to regulate hair growth and development. Hence, *FGF5* editing maintains DPCs in better condition to help hair follicle growth.

The growth of the mammalian coat may be the result of many signaling pathways and hormones acting in concert through certain signaling pathways, and inappropriate activation of these signals may result in abnormalities in the hair follicle cycle, resulting in hair loss and skin diseases [28,29]. FGF5 is a key factor in regulating hair growth. Notably, according to the genome-wide association studies, FGF5 polymorphism is significantly associated with hypertension susceptibility [30,31,32], and high levels of cortisol are the main causes of hypercortisolism. In skin, GC plays a crucial role in homeostasis and stress responses by the GR. The mammalian skin and hair have the same system as the hypothalamic-pituitary-adrenal axis (HPA) to react to stress and also synthesize GCs through their own neuroendocrine systems. The HPA axis has been associated with hair loss [33]. Besides, previous studies have shown that corticotropin-releasing hormone substantially inhibited hair growth, induced hair regression, inhibited hair matrix keratinocyte proliferation, and induced keratinocyte apoptosis [34]. Therefore, hair-follicle-derived cortisol may mediate paracrine or endocrine influences on hair follicles. It is reasonable to hypothesize that expression of the *FGF5* gene also affects cortisol levels in the skin. We found that *FGF5* editing reduced cortisol levels in the skin as well as decreased the expression of the GR, suggesting that *FGF5* editing can alleviate stress. It has been reported that the proliferation of keratinocytes is reduced in GC-treated murine skin, and GRs are effective inhibitors of epidermal proliferation [35,36]. Under chronic stress conditions, corticosterone acts on the DP to suppress the expression of Gas6 and keep the hair follicle in a prolonged resting period [37]. In our study, *FGF5* editing induced changes in cortisol levels, like decreased expression of cortisol and GR, eventually inducing hair follicle growth.

Oxidative stress not only affects animal health and production performance, but also inhibits animal economic performance. Hormones related to HPA (corticotropin-releasing hormone, adrenocorticotropic hormone, and cortisol) and potent antioxidants respond to local damage in the skin. In addition, dexamethasone (Dex), a synthetic GC, induces excessive oxidative stress that negatively affects osteoblast differentiation [38,39]. Previous studies have suggested that the oxidative stress during secondary follicle development may be the main cause of inhibition of secondary follicle development. Yang et al. reported that melatonin supplementation promoted the initiation and maturation of secondary follicles and increased their number in cashmere goat kids, which also enhanced the activity of antioxidant enzymes such as GSH-Px and T-AOC [40]. In addition, oxidative stress and inflammatory responses are considered to be associated with hair loss [41,42]. Lipid peroxides in oxidative stress induced hair follicle cell apoptosis and led to the early onset of the catagen phase in murine hair cycles, while hair growth was restored to normal with the addition of antioxidants [43]. In the present study, we revealed that an increase in the antioxidant enzyme activities of T-AOC, CAT, and GSH-Px in the GE group reduces oxidative damage.

The Wnt signaling pathway is a key factor in hair morphogenesis and hair cycle progression [44]. For example, activated β-catenin binds to the LEF/TCF transcription factors in the nucleus to transactivate downstream target genes to promote hair growth [45]. Overexpression of Wnt ligands in the epidermis increases the number of regenerated hair follicles and plays a key role in epidermal stem cell activation [46,47]. Besides, DP cells need to activate this pathway to maintain hair induction activity, thereby prolonging the anagen phase [48]. The current research also found that the Wnt/β-catenin signaling pathway is expressed in sheep skin at the beginning of SHF development [49]. Notably, *FGF5* editing significantly increased fine wool and active hair follicle density by increasing Wnt/β-catenin signaling [17]. RSPOs are a group of secreted proteins that enhance Wnt/β-catenin signaling. As shown in previous studies, by inhibiting the Znrf3/Rnf43 complex of Wnt signaling pathways in the hair matrix, the Rspo family forms a positive feedback loop of Wnt signaling to maintain the anagen phase of hair follicles [50,51]. As expected, we further showed that *FGF5* editing upregulated the Rspo family, whereas it downregulated Notum and DKK2, thereby activating growth factor HGF, a downstream target gene of Wnt signaling. Alteration of Rspo3 and Notum in the current study suggested that Wnt activation was the key to regulating *FGF5* editing-mediated fine wool growth.

**Figure 10:**
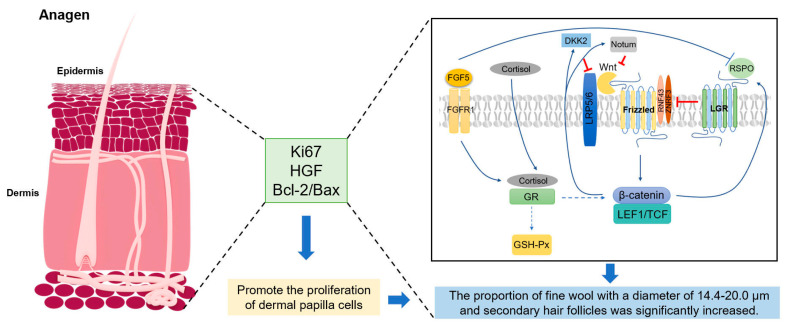
Mechanisms of *FGF5* promoting secondary hair follicle growth. *FGF5* editing promotes fine wool growth by reducing cortisol concentration, increasing the antioxidant enzyme activity, and activating the Wnt pathway. Abbreviations: HGF, hepatocyte growth factor; FGF5, fibroblast growth factor 5; FGFR1, FGF receptor 1; GR, glucocorticoid receptor; LGR, leucine-rich repeat-containing G-protein-coupled receptor; RSPO, Rspondin; Wnt, Wingless-type; LRP, LD-related protein.

In summary, we found that *FGF5* editing significantly downregulated cortisol levels, promoted the increase of fine wool density, and increased the proportion of fine wool with a diameter of 14.4–20.0 μm. The downregulation of cortisol also increases antioxidant enzyme activity and activates the Wnt signaling pathway, thereby promoting SHF proliferation growth (Figure 10). Our findings provide a new theoretical basis for the molecular breeding of fine wool sheep production. Moreover, the morphological development of sheep hair follicles is similar to that of human hair follicles, and their structural features allow them to be easily used as a model for studying human hair and skin. Therefore, the role of FGF5 in hormonal hair loss (like telogen effluvium, androgenetic alopecia, and AA) can be studied using this model.

## Data Availability

The data analyzed during the current study are available from the corresponding author on reasonable request.

## Supplementary Materials

The following supporting information can be downloaded at: https://www.mdpi.com/article/10.3390/cells13110985/s1, Table S1: Primers for qPCR; Figure S1: Proportion statistics of non-medullated wool, heterotypical wool, and medullated wool during anagen phase; Figure S2: Detection of wool density during telogen; Figure S3: One-month wool stretched length of GE sheep and WT sheep; Figure S4: The wool yield of GE and WT sheep; Figure S5: The clean wool yield and wool grease rate in GE and WT sheep; Figure S6: Skin tissue structural parameters in the GE and WT groups.

## References

[1] Schneider M.R., Schmidt-Ullrich R., Paus R. (2009). The hair follicle as a dynamic miniorgan. Curr. Biol. CB.

[2] Oh J.W., Kloepper J., Langan E.A., Kim Y., Yeo J., Kim M.J., Hsi T.C., Rose C., Yoon G.S., Lee S.J. (2016). A Guide to Studying Human Hair Follicle Cycling In Vivo. J. Investig. Dermatol..

[3] Belov A.A., Mohammadi M. (2013). Molecular mechanisms of fibroblast growth factor signaling in physiology and pathology. Cold Spring Harb. Perspect. Biol..

[4] Suzuki S., Ota Y., Ozawa K., Imamura T. (2000). Dual-mode regulation of hair growth cycle by two Fgf-5 gene products. J. Investig. Dermatol..

[5] Hébert J.M., Rosenquist T., Götz J., Martin G.R. (1994). FGF5 as a regulator of the hair growth cycle: Evidence from targeted and spontaneous mutations. Cell.

[6] Haub O., Goldfarb M. (1991). Expression of the fibroblast growth factor-5 gene in the mouse embryo. Development.

[7] Sundberg J.P., Rourk M.H., Boggess D., Hogan M.E., Sundberg B.A., Bertolino A.P. (1997). Angora mouse mutation: Altered hair cycle, follicular dystrophy, phenotypic maintenance of skin grafts, and changes in keratin expression. Vet. Pathol..

[8] Drögemüller C., Rüfenacht S., Wichert B., Leeb T. (2007). Mutations within the FGF5 gene are associated with hair length in cats. Anim. Genet..

[9] Cadieu E., Neff M.W., Quignon P., Walsh K., Chase K., Parker H.G., Vonholdt B.M., Rhue A., Boyko A., Byers A. (2009). Coat variation in the domestic dog is governed by variants in three genes. Science.

[10] Legrand R., Tiret L., Abitbol M. (2014). Two recessive mutations in FGF5 are associated with the long-hair phenotype in donkeys. Genet. Sel. Evol. GSE.

[11] Higgins C.A., Petukhova L., Harel S., Ho Y.Y., Drill E., Shapiro L., Wajid M., Christiano A.M. (2014). FGF5 is a crucial regulator of hair length in humans. Proc. Natl. Acad. Sci. USA.

[12] Li G., Zhou S., Li C., Cai B., Yu H., Ma B., Huang Y., Ding Y., Liu Y., Ding Q. (2019). Base pair editing in goat: Nonsense codon introgression into FGF5 results in longer hair. FEBS J..

[13] Cain D.W., Cidlowski J.A. (2017). Immune regulation by glucocorticoids. Nat. Rev. Immunol..

[14] Oakley R.H., Cidlowski J.A. (2013). The biology of the glucocorticoid receptor: New signaling mechanisms in health and disease. J. Allergy Clin. Immunol..

[15] Cascallana J.L., Bravo A., Donet E., Leis H., Lara M.F., Paramio J.M., Jorcano J.L., Pérez P. (2005). Ectoderm-targeted overexpression of the glucocorticoid receptor induces hypohidrotic ectodermal dysplasia. Endocrinology.

[16] Paus R., Handjiski B., Czarnetzki B.M., Eichmüller S. (1994). A murine model for inducing and manipulating hair follicle regression (catagen): Effects of dexamethasone and cyclosporin A. J. Investig. Dermatol..

[17] Zhang R., Li Y., Jia K., Xu X., Li Y., Zhao Y., Zhang X., Zhang J., Liu G., Deng S. (2020). Crosstalk between androgen and Wnt/β-catenin leads to changes of wool density in FGF5-knockout sheep. Cell Death Dis..

[18] Hu R., Fan Z.Y., Wang B.Y., Deng S.L., Zhang X.S., Zhang J.L., Han H.B., Lian Z.X. (2017). RAPID COMMUNICATION: Generation of FGF5 knockout sheep via the CRISPR/Cas9 system. J. Anim. Sci..

[19] Ota Y., Saitoh Y., Suzuki S., Ozawa K., Kawano M., Imamura T. (2002). Fibroblast growth factor 5 inhibits hair growth by blocking dermal papilla cell activation. Biochem. Biophys. Res. Commun..

[20] Harshuk-Shabso S., Dressler H., Niehrs C., Aamar E., Enshell-Seijffers D. (2020). Fgf and Wnt signaling interaction in the mesenchymal niche regulates the murine hair cycle clock. Nat. Commun..

[21] Hu X., Hao F., Li X., Xun Z., Gao Y., Ren B., Cang M., Liang H., Liu D. (2021). Generation of VEGF knock-in Cashmere goat via the CRISPR/Cas9 system. Int. J. Biol. Sci..

[22] Wang X., Cai B., Zhou J., Zhu H., Niu Y., Ma B., Yu H., Lei A., Yan H., Shen Q. (2016). Disruption of FGF5 in Cashmere Goats Using CRISPR/Cas9 Results in More Secondary Hair Follicles and Longer Fibers. PLoS ONE.

[23] Rosenquist T.A., Martin G.R. (1996). Fibroblast growth factor signalling in the hair growth cycle: Expression of the fibroblast growth factor receptor and ligand genes in the murine hair follicle. Dev. Dyn. Off. Publ. Am. Assoc. Anat..

[24] Stenn K.S., Paus R. (2001). Controls of hair follicle cycling. Physiol. Rev..

[25] Myung P.S., Takeo M., Ito M., Atit R.P. (2013). Epithelial Wnt ligand secretion is required for adult hair follicle growth and regeneration. J. Investig. Dermatol..

[26] Bull J.J., Müller-Röver S., Patel S.V., Chronnell C.M., McKay I.A., Philpott M.P. (2001). Contrasting localization of c-Myc with other Myc superfamily transcription factors in the human hair follicle and during the hair growth cycle. J. Investig. Dermatol..

[27] Rahmani W., Abbasi S., Hagner A., Raharjo E., Kumar R., Hotta A., Magness S., Metzger D., Biernaskie J. (2014). Hair follicle dermal stem cells regenerate the dermal sheath, repopulate the dermal papilla, and modulate hair type. Dev. Cell.

[28] Kulessa H., Turk G., Hogan B.L. (2000). Inhibition of Bmp signaling affects growth and differentiation in the anagen hair follicle. EMBO J..

[29] Ito T. (2010). Hair follicle is a target of stress hormone and autoimmune reactions. J. Dermatol. Sci..

[30] Liu C., Li H., Qi Q., Lu L., Gan W., Loos R.J., Lin X. (2011). Common variants in or near FGF5, CYP17A1 and MTHFR genes are associated with blood pressure and hypertension in Chinese Hans. J. Hypertens..

[31] Newton-Cheh C., Johnson T., Gateva V., Tobin M.D., Bochud M., Coin L., Najjar S.S., Zhao J.H., Heath S.C., Eyheramendy S. (2009). Genome-wide association study identifies eight loci associated with blood pressure. Nat. Genet..

[32] Takeuchi F., Isono M., Katsuya T., Yamamoto K., Yokota M., Sugiyama T., Nabika T., Fujioka A., Ohnaka K., Asano H. (2010). Blood pressure and hypertension are associated with 7 loci in the Japanese population. Circulation.

[33] Zhang X., Yu M., Yu W., Weinberg J., Shapiro J., McElwee K.J. (2009). Development of alopecia areata is associated with higher central and peripheral hypothalamic-pituitary-adrenal tone in the skin graft induced C3H/HeJ mouse model. J. Investig. Dermatol..

[34] Arck P.C., Handjiski B., Hagen E., Joachim R., Klapp B.F., Paus R. (2001). Indications for a ‘brain-hair follicle axis (BHA)’: Inhibition of keratinocyte proliferation and up-regulation of keratinocyte apoptosis in telogen hair follicles by stress and substance P. FASEB J. Off. Publ. Fed. Am. Soc. Exp. Biol..

[35] Demerjian M., Choi E.H., Man M.Q., Chang S., Elias P.M., Feingold K.R. (2009). Activators of PPARs and LXR decrease the adverse effects of exogenous glucocorticoids on the epidermis. Exp. Dermatol..

[36] Budunova I.V., Kowalczyk D., Pérez P., Yao Y.J., Jorcano J.L., Slaga T.J. (2003). Glucocorticoid receptor functions as a potent suppressor of mouse skin carcinogenesis. Oncogene.

[37] Choi S., Zhang B., Ma S., Gonzalez-Celeiro M., Stein D., Jin X., Kim S.T., Kang Y.L., Besnard A., Rezza A. (2021). Corticosterone inhibits GAS6 to govern hair follicle stem-cell quiescence. Nature.

[38] Zhen Y.F., Wang G.D., Zhu L.Q., Tan S.P., Zhang F.Y., Zhou X.Z., Wang X.D. (2014). P53 dependent mitochondrial permeability transition pore opening is required for dexamethasone-induced death of osteoblasts. J. Cell. Physiol..

[39] Chen J., Liang J.Q., Zhen Y.F., Chang L., Zhou Z.T., Shen X.J. (2021). DCAF1-targeting microRNA-3175 activates Nrf2 signaling and inhibits dexamethasone-induced oxidative injury in human osteoblasts. Cell Death Dis..

[40] Yang C.H., Xu J.H., Ren Q.C., Duan T., Mo F., Zhang W. (2019). Melatonin promotes secondary hair follicle development of early postnatal cashmere goat and improves cashmere quantity and quality by enhancing antioxidant capacity and suppressing apoptosis. J. Pineal Res..

[41] Haslam I.S., Jadkauskaite L., Szabó I.L., Staege S., Hesebeck-Brinckmann J., Jenkins G., Bhogal R.K., Lim F.L., Farjo N., Farjo B. (2017). Oxidative Damage Control in a Human (Mini-) Organ: Nrf2 Activation Protects against Oxidative Stress-Induced Hair Growth Inhibition. J. Investig. Dermatol..

[42] Saceda-Corralo D., Pindado-Ortega C., Moreno-Arrones O.M., Ortega-Quijano D., Fernández-Nieto D., Jiménez-Cauhe J., Vañó-Galván S. (2020). Association of Inflammation With Progression of Hair Loss in Women With Frontal Fibrosing Alopecia. JAMA Dermatol..

[43] Naito A., Midorikawa T., Yoshino T., Ohdera M. (2008). Lipid peroxides induce early onset of catagen phase in murine hair cycles. Int. J. Mol. Med..

[44] Ito M., Yang Z., Andl T., Cui C., Kim N., Millar S.E., Cotsarelis G. (2007). Wnt-dependent de novo hair follicle regeneration in adult mouse skin after wounding. Nature.

[45] DasGupta R., Fuchs E. (1999). Multiple roles for activated LEF/TCF transcription complexes during hair follicle development and differentiation. Development.

[46] Hawkshaw N.J., Hardman J.A., Alam M., Jimenez F., Paus R. (2020). Deciphering the molecular morphology of the human hair cycle: Wnt signalling during the telogen-anagen transformation. Br. J. Dermatol..

[47] Clevers H., Loh K.M., Nusse R. (2014). Stem cell signaling. An integral program for tissue renewal and regeneration: Wnt signaling and stem cell control. Science.

[48] Kishimoto J., Burgeson R.E., Morgan B.A. (2000). Wnt signaling maintains the hair-inducing activity of the dermal papilla. Genes Dev..

[49] Gao Y., Wang X., Yan H., Zeng J., Ma S., Niu Y., Zhou G., Jiang Y., Chen Y. (2016). Comparative Transcriptome Analysis of Fetal Skin Reveals Key Genes Related to Hair Follicle Morphogenesis in Cashmere Goats. PLoS ONE.

[50] Hao H.X., Xie Y., Zhang Y., Charlat O., Oster E., Avello M., Lei H., Mickanin C., Liu D., Ruffner H. (2012). ZNRF3 promotes Wnt receptor turnover in an R-spondin-sensitive manner. Nature.

[51] Jiang X., Charlat O., Zamponi R., Yang Y., Cong F. (2015). Dishevelled promotes Wnt receptor degradation through recruitment of ZNRF3/RNF43 E3 ubiquitin ligases. Mol. Cell.

