# Supplementary material for: Increasing GSH-Px Activity and Activating Wnt Pathway Promote Fine Wool Growth in FGF5-Edited Sheep
Source: Cells. 2024 Jun 5;13(11):985. doi: 10.3390/cells13110985 (PMC11172217; doi:10.3390/cells13110985)

Table S1 Primers for qPCR

Genes	Accession	Primer sequence (5’-3’)	Product size (bp)	T_m (°C)
FGF5-1	NM_001246263.2	Forward: TGTGTCTCAGGGGATTGTAGG<br>Reverse: GGCGGAGGCATAGGTATTATAG	157	60
FGF5-2	NM_001246263.2	Forward: CACGTCTCTACCCACTTTCTG<br>Reverse: TTAGGACTTCTCCGAGGTGC	143	60
FGFR1	XM_015104616.1	Forward: CAAACCAAACCGTATGCCCG<br>Reverse: ACCTTGTAGCCTCCGATCCT	192	60
GR	>NM_001114186.1	Forward: CCACCTCCCAAACTCTGCCTAG<br>Reverse: TGCTGTCCTTCCACTGCTCTT	113	60
Ki67	>XM_042238739.1	Forward: GAGGCCAATTTGATCGTGGC<br>Reverse: AGTGTTGGCTCTTCTCTGCC	129	60
HGF	>XM_004007806.5	Forward: AGGTACGCTACGAAGTCTGTGA<br>Reverse: CGGTGTGGTGTCTGATGATCC	142	60
Bcl2	>XM_012103831.4	Forward: CCTGTGGATGACCGAGTACCTG<br>Reverse: CCAGACTGAGCAGTGCCTTCA	149	60
Bax	>XM_027978592.2	Forward: TCATGGGCTGGACATTGGACTT<br>Reverse: CAGATGGTGAGTGAGGCAGTGA	157	60
RSPO1	>XM_042245742.1	Forward: GAGGAACGACATCCGCCAAG<br>Reverse: GCCTCGCAGTGCTCAATCTT	117	60
RSPO2	>XM_015097839.3	Forward: GTGTGGAAGGATGTGAGGTTGG<br>Reverse: TGGATTCAGCAATGGTTGGACA	159	60
RSPO3	>XM_004011136.4	Forward: CCAGTGAGGCGAGGAAGTGTA<br>Reverse: GGGCTCCAGACCTTTGTTGTC	143	60
RSPO4	>XM_004014778.4	Forward: CACTCTGCCTGTTGCTGCTTG<br>Reverse: GGATACCTTCCCGACGGATGAA	180	60
LRP5	>XM_027959820.2	Forward: CGACGGCTCTGACGAACTCA<br>Reverse: CGCTGGCACACGAAGTAGAC	141	60
LRP6	>XM_042247517.1	Forward: ATCCGCCGCTCCTTCATAGAT<br>Reverse: TATCCGATCAGTGCCAGTGTCT	132	60
Axin2	>XM_042256527.1	Forward: CAACAGCATCGTCTCCAAGCA<br>Reverse: GGTAGGCGTTCTCCTCCATCA	140	60
LEF1	>XM_042251151.1	Forward: AAGGAACAGCAGCAGACTCAGT<br>Reverse: CAACGACGCAGTCAGCAACA	106	60
Notum	>XM_042256238.1	Forward: TGGAATGCCAACATGGTCTTCA<br>Reverse: TCGCACAACCTCCTGGATGA	123	60
DKK2	>XM_004009640.5	Forward: TTGCTGTGCTCGTCACTTCTG<br>Reverse: TTGGAGGAGTAGGTGGCATCTT	171	60
CCND1	>XM_027959928.2	Forward: GCCGAGGAGAACAAGCAGATC<br>Reverse: TGATAGGAGAGGAAGCCGTTGG	170	60
c-Myc	>XM_042253807.1	Forward: GGACACGGAGGAGAATGACAAG<br>Reverse: TGGACCGACAGGATGTATGCT	177	60

GADPH	NM_001190390.1	Forward: CTGACCTGCCGCCTGGAGAAA<br>Reverse: GTAGAAGAGTGAGTGTCGCTGTT	149	60

Figure S1 Proportion statistics of non-medullated wool, heterotypical wool, and medullated wool during anagen phase: (A) Proportion statistics of non-medullar wool in the three parts; (B) Proportion statistics of heterotypical wool in the three parts; (C) Proportion statistics of medullated wool in the three parts.

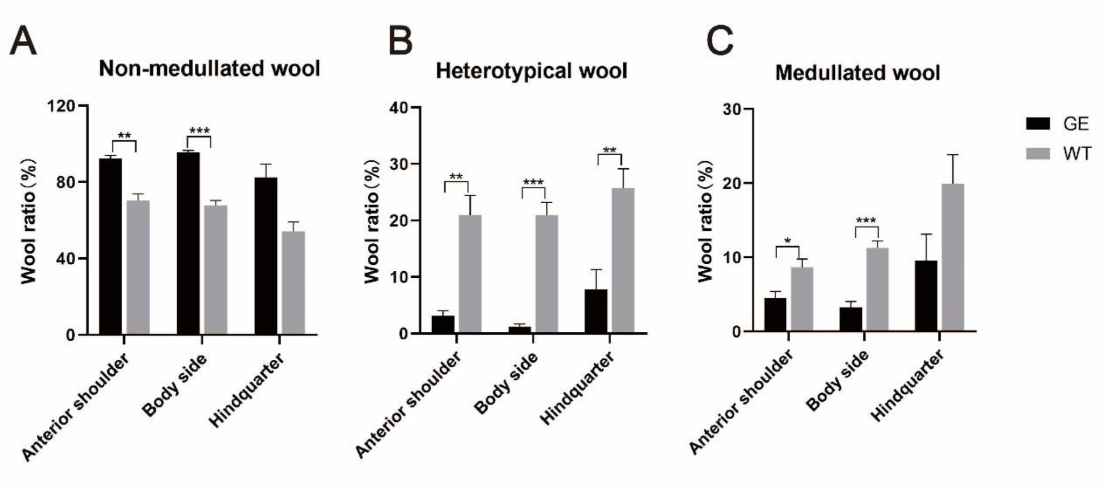

Figure S2 Detection of wool density during telogen: (A) Photograph of skin follicles 48 h after shaving. The actual area shown in the picture is 9 mm². (B) Changes in coarse and fine wool density in different parts of GE and control group. The statistical results in figure B are from figure A.

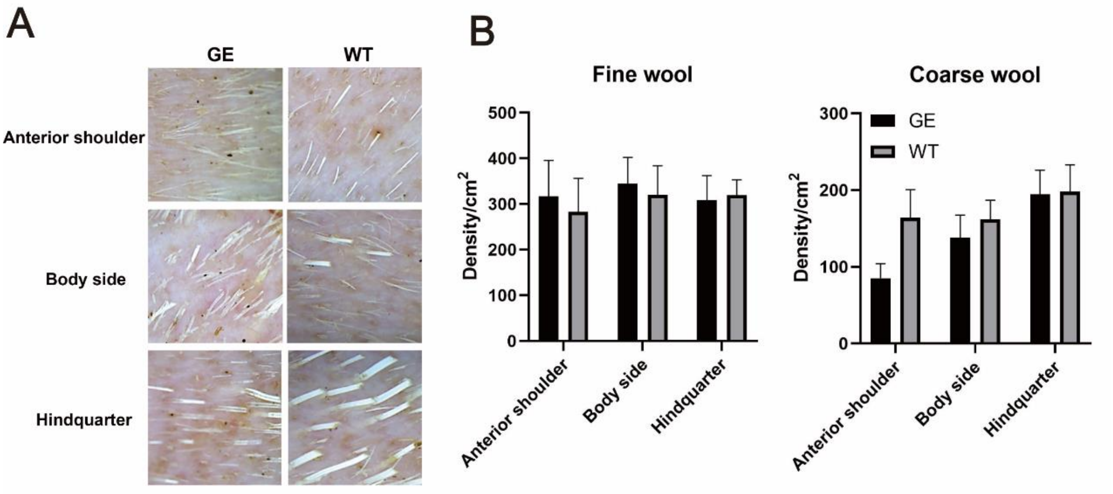

Figure S3 One-month wool stretched length of GE sheep and WT sheep: (A) The stretched length of fine wool; (B) The stretched length of coarse wool.

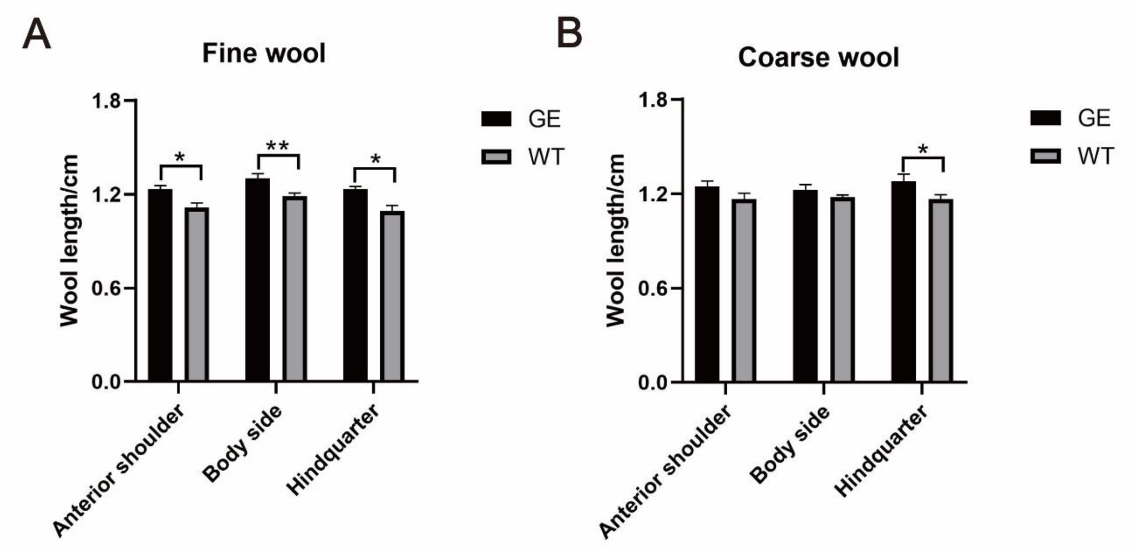

Figure S4 The wool yield of GE and WT sheep.

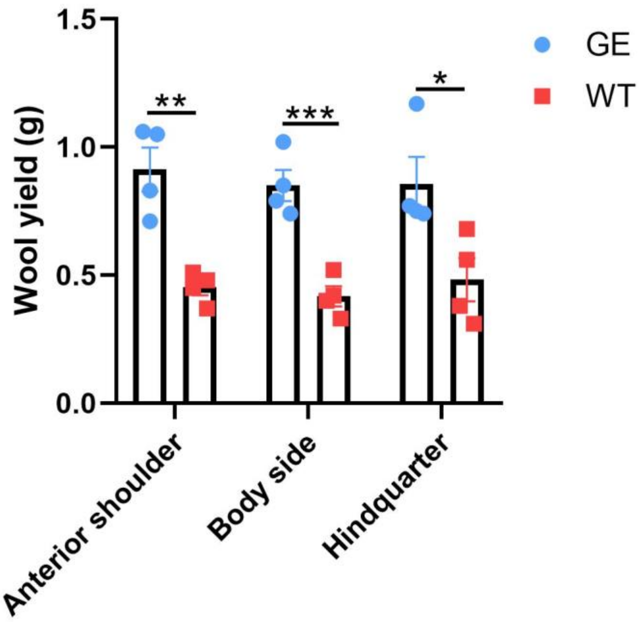

Figure S5 The clean wool yield and wool grease rate in GE and WT sheep: (A) The clean wool yield of GE and WT sheep; (B) The wool grease rate of GE and WT sheep.

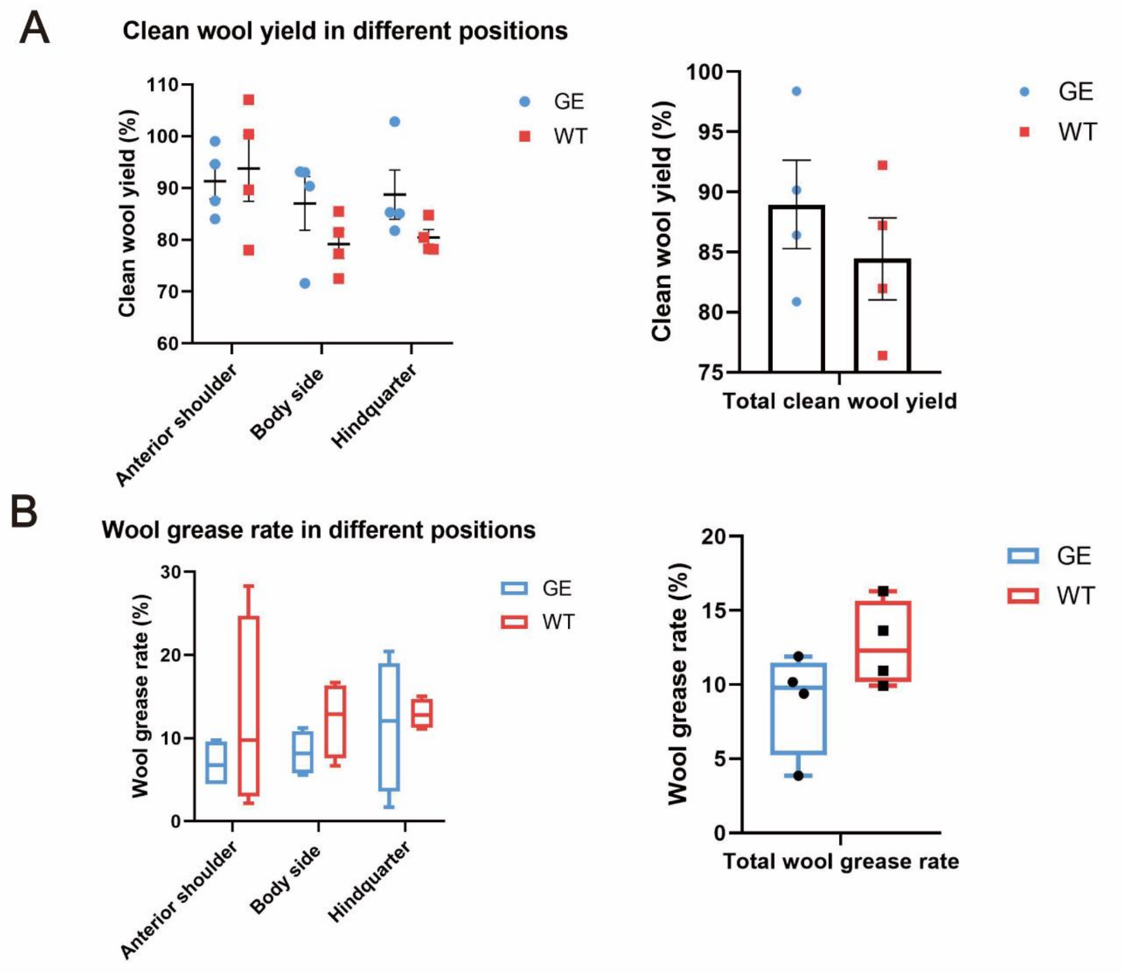

Figure S6 Skin tissue structural parameters in the GE and WT groups: (A) Primary hair follicle diameter statistics of GE and WT; (B) The depth of primary hair follicle in GE and WT groups; (C) The depth of secondary follicle in GE and WT groups.

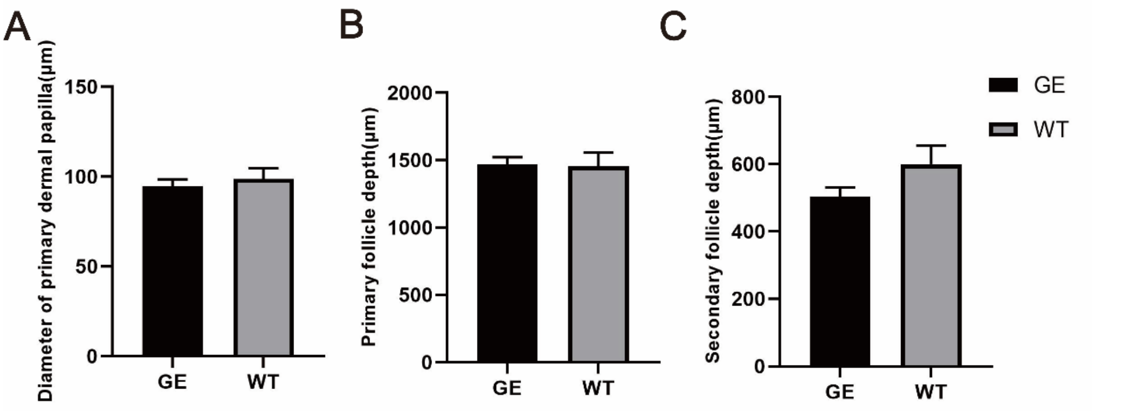

Supplement: Supplementary file 1 [file cells-13-00985-s001.zip › cells-2986877-supplementary.pdf]
